# Compressive Spectral Method for the Simulation of the Nonlinear Gravity Waves

**DOI:** 10.1038/srep22100

**Published:** 2016-02-25

**Authors:** Cihan Bayındır

**Affiliations:** 1 Işık University, Department of Civil Engineering, Şile, 34980, Istanbul, Turkey

## Abstract

In this paper an approach for decreasing the computational effort required for the spectral simulations of the fully nonlinear ocean waves is introduced. The proposed approach utilizes the compressive sampling algorithm and depends on the idea of using a smaller number of spectral components compared to the classical spectral method. After performing the time integration with a smaller number of spectral components and using the compressive sampling technique, it is shown that the ocean wave field can be reconstructed with a significantly better efficiency compared to the classical spectral method. For the sparse ocean wave model in the frequency domain the fully nonlinear ocean waves with Jonswap spectrum is considered. By implementation of a high-order spectral method it is shown that the proposed methodology can simulate the linear and the fully nonlinear ocean waves with negligible difference in the accuracy and with a great efficiency by reducing the computation time significantly especially for large time evolutions.

The signals, with majority of the components are zero, are called sparse signals. Like majority of the signals in the nature, the ocean waves are sparse either in the time or in the frequency domain. Therefore the compressive sampling technique can be thought as a very efficient tool for measuring or simulating the ocean waves. In this paper it is shown that the efficiency of the compressive sampling technique can also be used for the improvement of the computational simulation efforts of the ocean waves.

The linear and fully nonlinear ocean gravity waves are considered in this study. Ocean waves are simulated by implementation of a high order spectral method in which the spectral derivatives are evaluated using the fast Fourier transforms (FFT) over the periodic domain. Time integration is carried out using a 4^*th*^ order Runge-Kutta method. The proposed approach, which can be named as compressive spectral method, depends on the idea of using smaller number of spectral components compared to the classical spectral method. Performing time integration with smaller number of spectral components and using the compressive sampling technique, it shown that the ocean wave field can be modeled with a significantly better efficiency compared to the classical spectral method. The sparsity property of the wave field in the frequency domain which has the Jonswap form is used and *l*_1_ minimization step of the compressive sampling algorithm is applied in the frequency domain.

It is shown that by using a smaller number of spectral components and the compressive sampling technique, ocean waves can be simulated very efficiently compared to the classical spectral simulation with a larger number of spectral components. Also it is shown that the accuracy difference between two models is of negligible importance. Therefore it is shown that the proposed compressive spectral method can be a very efficient tool in simulations of fully nonlinear ocean waves.

## Methodology

### Review of a Linear Ocean Wave Model

Many different approximate equations are developed to model the ocean waves. Also there are various models which solve the fully nonlinear kinematic and dynamic boundary conditions^1^. In order to discuss the efficiency of the proposed technique first linear ocean waves are considered. Linearized kinematic and dynamic boundary conditions for the ocean waves at *z* = 0 are given as


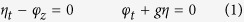


where *η* and *φ* denote the water surface fluctuation and the velocity potential, respectively^1^. Although this set of equations can be solved analytically to yield sinusoidal waveforms, in order to discuss the advantages of the compressive sampling technique, a numerical spectral method is implemented for simulating the linear ocean waves. In a periodic domain with arbitrary depth *h*, the velocity potential *φ* can be expressed by


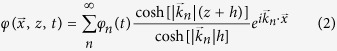


where 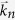 is the wave-number vector^1^,^2^,^3^. *φ*_*z*_ at *z* = 0 can be written as


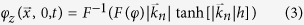


where *F* and *F*^−1^ denote the Fourier and inverse Fourier transformations respectively. In this paper only one dimensional waves are studied.

This set of equations are solved with a 4^*th*^ order Runge-Kutta method for time integration in order to simulate the linear ocean waves for various initial linear ocean wave profiles. Initial linear ocean wave fields are constructed by an inverse FFT algorithm using random spectral components with total energy described by the Jonswap spectrum. Details and further discussion can be seen in^1^.

### Review of Wave Model for Fully Nonlinear Waves

The high order spectral method summarized in this section is first presented in^4^; which models the nonlinear ocean surface waves on the surface of an ideal fluid. An implementation of this model both in 1D and 2D can be seen in^1^,^2^,^3^,^5^.

Following^6^, the velocity potential at the ocean surface is denoted by 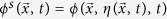 where *η* is the water surface fluctuation and *ϕ* is the velocity potential. Using *ϕ*^*s*^, it is possible to show that potential flow of water waves on an deep fluid can be formulated as a canonical Hamiltonian system;


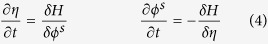


where *H* is the Hamiltonian which is the total energy of the wavefield. The details of this derivation can be seen in^6^. Employing the transformations


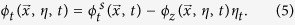


and


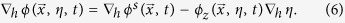


the classical kinematic and dynamic boundary conditions can be written as^1^


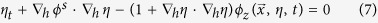


and


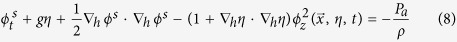


where *P*_*a*_ is the atmospheric pressure, *ρ* is the density of water and the horizontal gradient is 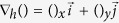. Using eigenfunction expansion, the velocity potential can be expressed as^1^,^4^


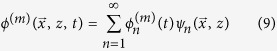


For deep water the shape function *ψ* takes the form of a decaying exponential so that^1^,^4^


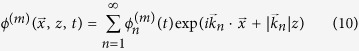


whereas for shallow water


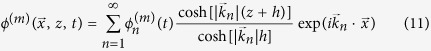


where *h* is the water depth^1^,^4^. Using Taylor series expansion about mean water level and perturbation series up to an arbitrary order *S*, the *ϕ*_*z*_ can be written as


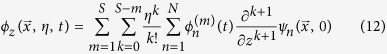


By utilizing the efficient FFTs on a periodic domain one can evaluate *ϕ*_*z*_ using (10–12). So that (7,8) can be solved numerically. In this study time integration is performed by a 4^*th*^ order Runge-Kutta method^7^, all spatial derivatives are calculated in the wavenumber domain and all multiplications for nonlinear terms are done in the physical space. Details of the numerical method used to solve this set of equations can be seen in^1^,^4^. Details of the numerical spectral methods can be seen in^8^,^9^.

### Review of the Compressive Sampling

After it has been introduced to the scientific community with a seminal paper^10^, compressive sampling (CS) has become a core research area in the last decade. Today it is widely used in various branches of applied mathematics, physics and engineering and some studies such as the development of a single pixel video camera system aim to make use of this efficient technique in digital systems as well. In summary, CS states that a sparse signal can be reconstructed from fewer samples than the samples that Nyquist-Shannon sampling theorem states. In this section we try to sketch a brief summary of the CS for the oceanographers.

Let *η* be a signal of length *N* and a *K*-sparse signal, that is only *K* out of *N* elements of the signal are nonzero. Using orthonormal basis functions with transformation matrix *ψ*, *η* can be represented in terms of basis functions. Typical transformation used today are the Fourier, wavelet or discrete cosine transforms just to mention few. Therefore it is possible to write 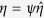 where 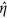 is the transformation coefficient vector. Discarding the zero coefficients of *η* which is a *K*-sparse signal one can obtain 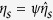 where *η*_*s*_ is the signal with non-zero components only.

The underlying idea of the CS is that a *K*-sparse signal *η* of length *N* can exactly be reconstructed from *M* ≥ *Cμ*^2^(*ξ*, *ψ*)*K* log(N) random measurements with a very high probability, where *C* is a positive constant and *μ*^2^(*ξ*, *ψ*) is the mutual coherence between the sensing basis *ξ* and transform basis *ψ*^10^. For the recovery of a sparse spectra the *M* samples are taken in physical domain or for the recovery of the sparse signal in the physical domain the *M* samples are taken in the spectra. Taking *M* random projections and by using the sensing matrix *ξ* one obtains *g* = *ξη*. Therefore the problem can be formulated as


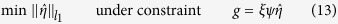


where 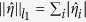. So that among all signal which satisfies the given constraints, the *l*_1_ solution of the CS problem is given as 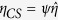.

*l*_1_ minimization is only one of the alternatives which can be used for this optimization problem. There are some other algorithms in literature to recover the sparse solutions such as reweighted *l*_1_ minimization or greedy pursuit algorithms^10^. Details of the CS can be seen in^10^,^11^.

### Proposed Methodology

In a classical spectral method let *N* be the number of the spectral components used for the representation of a signal. *N* is generally desired to be as large as possible that the computational effort allows. Here we introduce the compressive spectral method which states that by using *M* spectral components with *M* << *N* and using the CS technique to construct the *N*-component signal at the last step of the time evolution from *M* components, it is possible to obtain a very efficient computational method especially for very long time evolutions. The selection of the *M* spectral components has to be done carefully depending on the width of the *K*-sparse wave profile in order to satisfy the lower limit of *M* = *O*(*K* log(*N*/*K*)) condition of the CS algorithm where *O* denotes the order of symbol. For example for a 10-sparse signal with 1000 elements, that is for *K* = 10 and *N* = 1000 one would need *M* = *O*(20) components for the exact recovery of the 10-sparse signal by the CS. If a smaller *M* is used then the exact recovery of the sparse signal may become impossible. If a higher *M* is used that exact recovery can be done but one starts to lose the undersampling ratio advantage of the CS.

The CS theory states that *M* samples has to be chosen randomly. However for the many of the phenomena we encounter in the oceanography we have the priori knowledge about which spectral components will be nonzero. Some examples are sideband waves with few nonzero spectral components, Jonswap spectrum with more energy in the smallers wavenumbers, triangular spectrum of the rational rogue wave solutions of the nonlinear Schrödinger equation^12^ just to name a few. Therefore in order to make a more accurate recovery of the sparse spectra with nonzero components located in the lower wavenumbers we take *M* equally spaced samples in the physical ocean surface. Our undersampling ratio becomes *r* = *N*/*M*. This deterministic uniform sampling causes *r* replicas to appear in the recovered spectrum by the CS. We only need and keep the energy in the smaller wavenumber components and filter out the *r* − 1 aliasing high wavenumber replicas of the spectrum.

Starting from the initial conditions, time stepping is performed for only selected *M* spectral components. After the time stepping, the *N* point signal is reconstructed from *M* components with the help of the *l*_1_ minimization technique of the CS theory. It is shown that the methodology offered in here can reduce the computational effort significantly compared to the classical spectral method with *N* components while the accuracy difference in the results is negligible. In order to ensure the stability of the schemes both for the classical and the proposed compressive spectral methods, a small *dt* must be selected to satisfy the CFL condition. For example for *L* = 500 *m*, *N* = 2048, *M* = 256 the *dt* = 10^−5^ value is selected. This strict requirement is imposed by the classical spectral method, not by the proposed compressive spectral method. That is *dx*_*N*_ = *L*/*N* is smaller than *dx*_*M*_ = *L*/*M*, so in order to satisfy CFL condition (upper bounded *dt*/*dx*), smaller *dt* must be selected for the classical method. The proposed method also relaxes this restriction.

A similar approach has been introduced by us and discussed in^13^,^14^ where the waves which are sparse in the time domain are considered however in this paper the sparsity property of the frequency spectrum, which is in Jonswap form, is used to test the proposed method for the simulation of the linear and the fully nonlinear ocean waves.

## Results

### Results for Linear Ocean Wave Simulations

**Figure 1:**
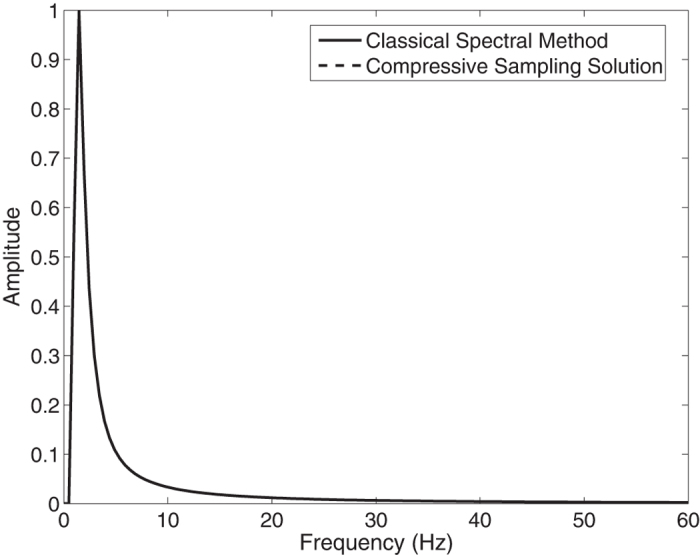
Comparison of the energy spectra of the classical spectral method and the proposed compressive spectral method for linear waves with *N* = 1024, *M* = 256.

In the Fig. 1, the Jonswap spectra of the *N* = 1024 component classical spectral method and the *M* = 256 component compressive spectral method proposed are compared. The two methods are in excellent agreement as it can be seen in the figure. The normalized root-mean-squared difference between two spectra is 0.0045 for this simulation.

**Figure 2:**
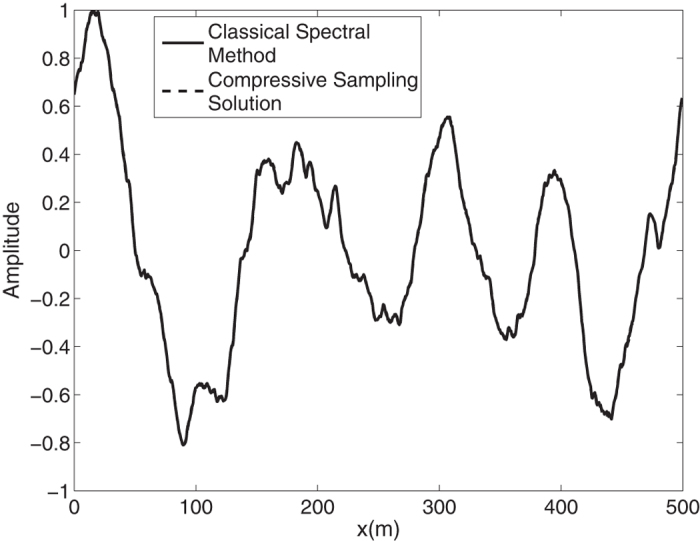
Comparison of the water surface fluctuation of the classical spectral method and the proposed compressive spectral method for linear waves with *N* = 1024, *M* = 256.

By means of an inverse FFT, it is possible to construct the ocean surface with linear waves which is not necessarily sparse. In the Fig. 2, the water surface fluctuations of the classical *N* = 1024 component spectral method and the *M* = 256 component compressive spectral method proposed are compared. The two methods are in excellent agreement as it can be seen in the figure. The normalized root-mean-square difference between two profiles is 0.0052 for this simulation.

**Figure 3:**
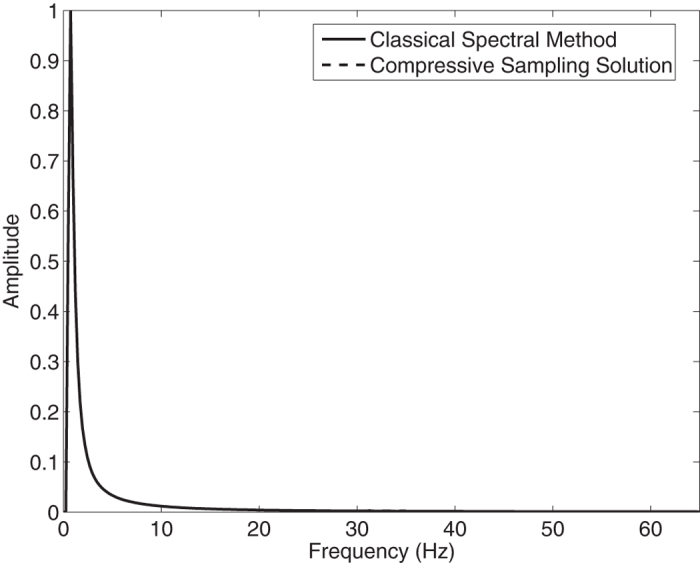
Comparison of the energy spectra of the classical spectral method and the proposed compressive spectral method for linear waves with *N* = 2048, *M* = 256.

In the Fig. 3, the Jonswap spectra of the *N* = 2048 component classical spectral method and the *M* = 256 component compressive spectral method proposed are compared. The two methods are in excellent agreement as it can be seen in the figure. The normalized root-mean-square difference between two spectra is 0.0044 for this simulation.

**Figure 4:**
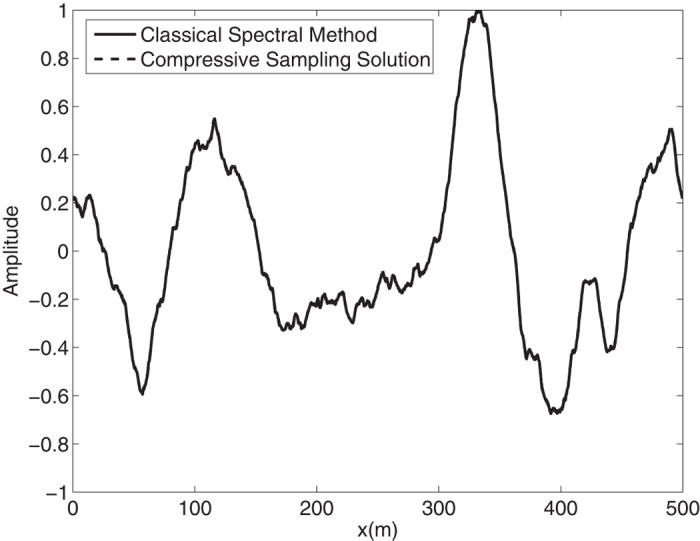
Comparison of the water surface fluctuation of the classical spectral method and the proposed compressive spectral method for linear waves with *N* = 2048, *M* = 256.

Again by means of an inverse FFT, it is possible to construct the ocean surface with linear waves which is not necessarily sparse. In the Fig. 4, the water surface fluctuations of the *N* = 1024 component classical spectral method and the *M* = 256 component compressive spectral method proposed are compared. Again the two methods are in excellent agreement in terms of accuracy. The normalized root-mean-square difference between two profiles is 0.0048 for this simulation.

**Figure 5:**
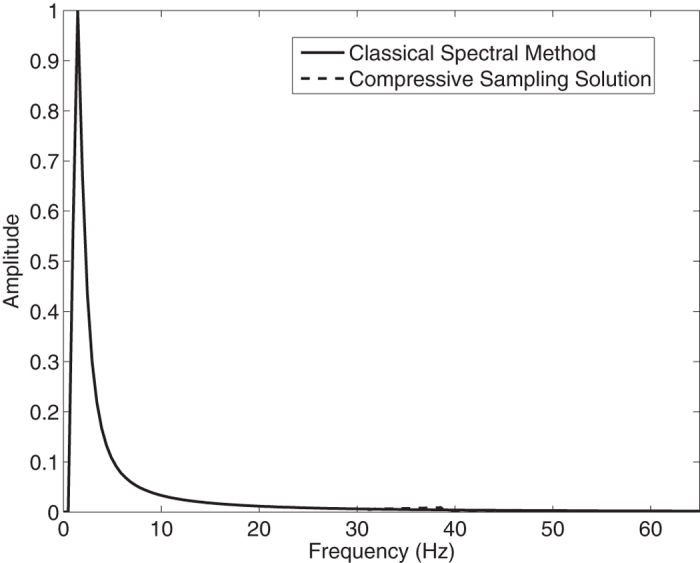
Comparison of the energy spectra of the classical spectral method and the proposed compressive spectral method for linear waves with *N* = 1024, *M* = 128.

The Jonswap spectra of the *N* = 1024 component classical spectral method and the *M* = 128 component compressive spectral method proposed are compared in the Fig. 5. The results of the two methods agrees well however it can be realized that using a smaller *M* causes the difference to increase although still it is of negligible importance. This is mainly due to the fact that for a smaller *M* the sparsity condition becomes critical. The normalized root-mean-square difference between two spectra is 0.0036 for this simulation.

**Figure 6:**
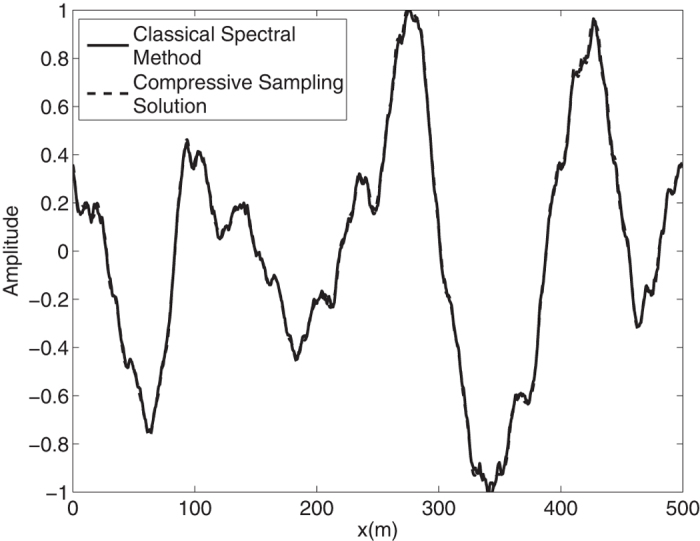
Comparison of the water surface fluctuation of the classical spectral method and the proposed compressive spectral method for linear waves with *N* = 1024, *M* = 128.

Again by means of an inverse FFT, it is possible to construct the ocean surface with linear waves which is not necessarily sparse. In the Fig. 6, the water surface fluctuations of the *N* = 1024 component classical spectral method and the *M* = 128 component compressive spectral method proposed are compared. The two methods are in very good agreement as it can be seen in the figure. The normalized root-mean-square difference between two profiles is 0.0165 for this simulation.

All of the results presented above show promising evidence for the accuracy of the proposed method for the linear ocean wave simulations. Additionally the computational efforts required to run the various configurations for the linear ocean wave simulations are summarized in the Table 1.

The average computation times of 50 realizations given in the Table 1 are in the units of seconds. The computation times are measured on a Dell Vostro 1700 laptop with dual core of 1.8 GHz and 1 GB RAM which is used to run the MATLAB code. As it can be seen on the table, for a very small number of time steps the compressive spectral method does not provide any improvement in the computational effort. This is mainly due to the computational effort required by the *l*_1_ minimization. However as the number of time steps gets bigger, the computational effort significantly reduces while the differences in the wave profiles are of negligible importance. Therefore compressive spectral method provides a great computational efficiency compared to the classical spectral method for the ocean wave simulations which are sparse in frequency domain.

### Results for Fully Nonlinear Monochromatic Waves

**Figure 7:**
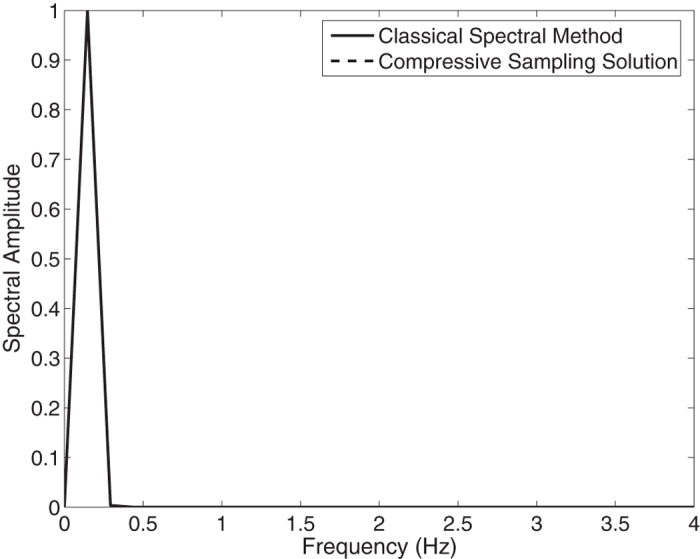
Comparison of classical spectral method and proposed compressive spectral method for the spectra of a monochromatic wave *N* = 1024, *M* = 256.

**Figure 8:**
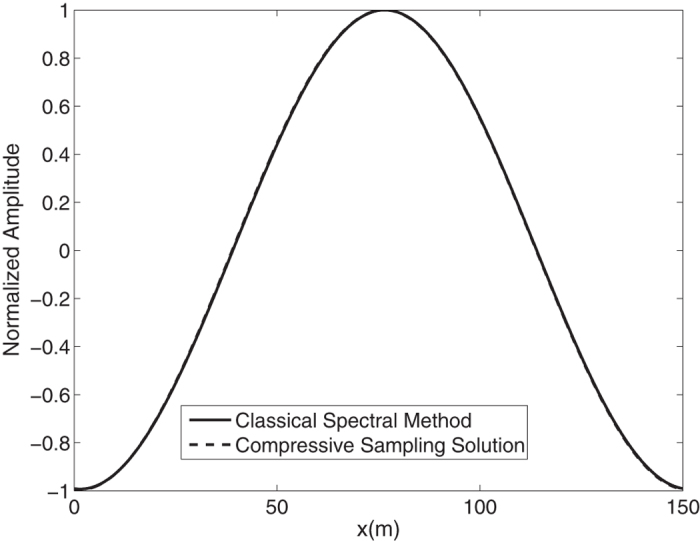
Comparison of classical spectral method and proposed compressive spectral method for a monochromatic wave *N* = 1024, *M* = 256.

In this section we solve the governing equations for the fully nonlinear model described above by the classical spectral method and by the proposed compressive spectral method for a monochromatic wave. In the Fig. 7, the spectra of the of the *N* = 1024 component classical spectral method and the *M* = 256 component compressive spectral method proposed are compared for a monochromatic wave. The two methods are in excellent agreement as it can be seen in the figure. The normalized root-mean-square difference between two profiles is 0.0230 for this simulation. Water surface fluctuation is obtained by taking the inverse FFT of this spectra and is shown in the Fig. 8 where the water surface fluctuations of the *N* = 1024 component classical spectral method and the *M* = 256 component compressive spectral method proposed are compared. The two methods are in excellent agreement as it can be seen in the figure. The normalized root-mean-square difference between two profiles is 0.0006 for this simulation.

### Results for Fully Nonlinear Trichromatic Waves (Sidebands)

**Figure 9:**
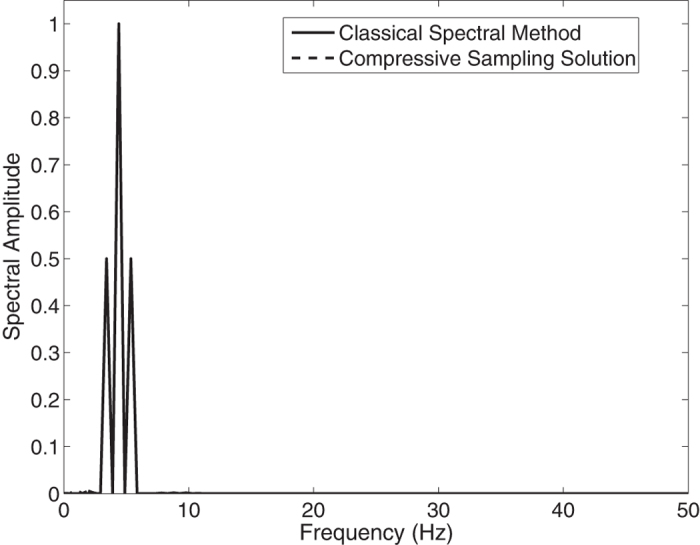
Comparison of classical spectral method and proposed compressive spectral method for a spectra with sidebands *N* = 1024, *M* = 256.

**Figure 10:**
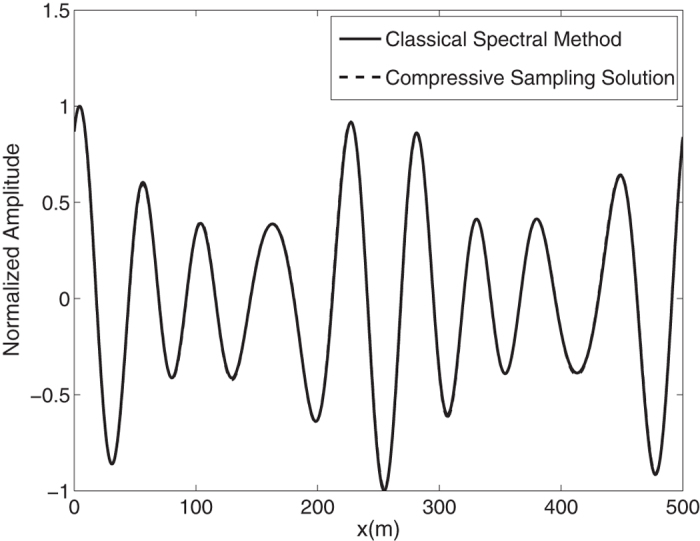
Comparison of classical spectral method and proposed compressive spectral method for a profile with one central wavenumber and two sidebands *N* = 1024, *M* = 256.

In this section we solve the governing equations for the fully nonlinear model described above by the classical spectral method and by the proposed compressive spectral method for a trichromatic wave. In the Fig. 9, the water surface fluctuations of the *N* = 1024 component classical spectral method and the *M* = 256 component compressive spectral method proposed are compared for a trichromatic profile, that is a wavefield with three wavenumber components. This is also known as the sidebands example, a wavefield with one central wavenumber component and two side neighbour components of this central wavenumber. The steepness for this surface is *ka* ≈ 0.1. The two methods are in excellent agreement as it can be seen in the figure. The normalized root-mean-square difference between two profiles is 0.0034 for this simulation. In the Fig. 10, the water surface fluctuations of the *N* = 1024 component classical spectral method and the *M* = 256 component compressive spectral method proposed are compared for the trichromatic wavefield. The normalized root-mean-square difference between two profiles is 0.0059 for this simulation and agreement is excellent.

**Figure 11:**
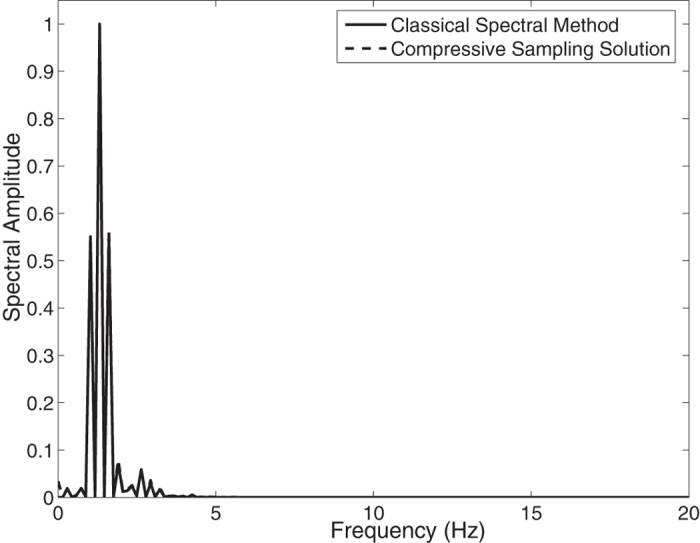
Comparison of classical spectral method and proposed compressive spectral method for a spectra with sidebands *N* = 1024, *M* = 256-nonlinear interactions.

**Figure 12:**
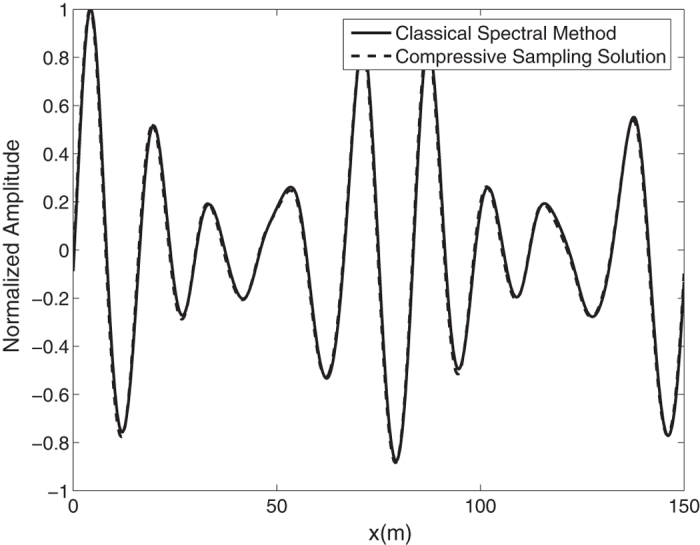
Comparison of classical spectral method and proposed compressive spectral method for a profile with one central wavenumber and two sidebands *N* = 1024, *M* = 256-nonlinear interactions.

As discussed in the review of compressive sampling section above, for a K-sparse frequency series at least *M* = *O*(*K* log(*N*/*K*)) samples are needed for the exact reconstruction. Therefore it is important to analyze how the proposed method will perform in the case of spectral broadening. For this purpose we consider the nonlinear wave interactions. We test the proposed method with longer temporal runs. Generally speaking, a time scale of 25*T*_*p*_ is sufficient for nonlinear interactions to be developed^5^,^15^ where *T*_*p*_ is the peak wave period. We test the method with a run time of 50*T*_*p*_. The resulting spectra for this simulation is presented in the Fig. 11 and the corresponding water surface fluctuation which is obtained by applying inverse FFT on this spectra can be seen in the Fig. 12. The normalized root-mean-square differences between two profiles are 0.0106 and 0.0073 for these two figures, respectively.

As it can be realized from the figures above, the method also works well in the case of spectral broadening due to nonlinear interactions. The spectra exhibits energy increase in higher and lower wavenumbers. For trichromatic waves, the *M* = *O*(*K* log(*N*/*K*)) condition of the compressive sampling is not strict because initially only three wavenumbers has energy and after the time stepping long enough to cause some interactions (for this example 50*T*_*p*_ is used) the energy has spread to only few other wavenumber components. Although there is spectral broadening, the spectra still satisfies the *M* = *O*(*K* log(*N*/*K*)) condition of the compressive sampling since *K* remains small, therefore the proposed compressive spectral method works well.

### Results for Fully Nonlinear Waves with Jonswap Spectrum

In this section we solve the governing equations for the fully nonlinear model described above by the classical spectral method and the proposed compressive spectral method for a wave field described by a Jonswap spectra. For small evolution times the results are no different than the results presented in linear wave simulations sections since nonlinear interaction do not develop within short time. Therefore we concentrate on runs with time scales long enough for the nonlinear interactions to be developed. However it is known that high order spectral method is subjected to high wavenumber instability in the form of a sawtooth function in the spatial domain which becomes more critical for longer runs. Therefore in order to obtain stable solutions smoothing filter must be applied. One possible form of the smoothing filter is given in^1^,^4^ and applied to *η* and *ϕ*^*s*^ in the Fourier domain. The effects of the smoothing filter such as the decay of the total wave energy is discussed in^1^,^4^. In compressive spectral method we propose smoothing filter is applied to *M* components and those components are the first *M* components of the classical smoothing filter with *N* terms.

**Figure 13:**
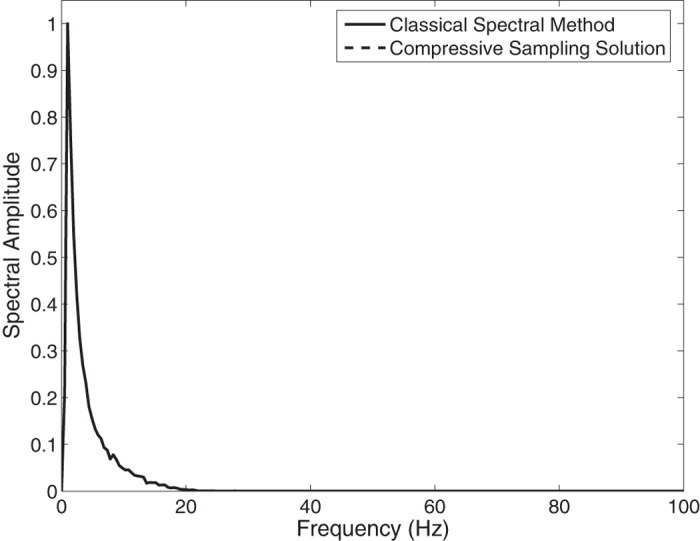
Comparison of classical spectral method and proposed compressive spectral method for a Jonswap spectrum with *N* = 2048, *M* = 256-nonlinear interactions.

**Figure 14:**
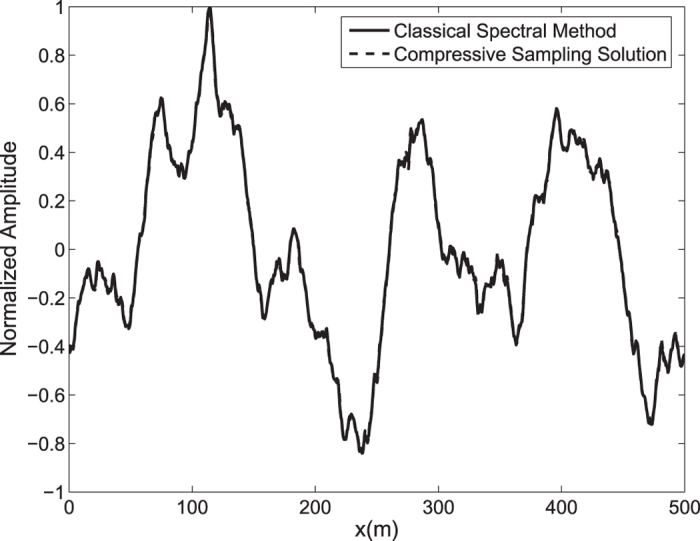
Comparison of classical spectral method and proposed compressive spectral method for a fully nonlinear periodic wave profile with *N* = 2048, *M* = 256-nonlinear interactions.

In the Fig. 13, the water surface fluctuations of the *N* = 2048 component classical spectral method and the *M* = 256 component compressive spectral method proposed are compared for a fully nonlinear ocean wave surface defined by a Jonswap spectrum. The two methods are in excellent agreement as it can be seen in the figure. The normalized root-mean-square difference between two profiles is 0.0099 for this simulation. The corresponding water surface fluctuation which is obtained by applying inverse FFT on for this spectra can be seen in the Fig. 14 with a normalized root-mean-square difference of 0.0104 between two profiles. As it can realized from the figures above and below, the method also works well in the case of spectral broadening due to nonlinear interactions. The spectra exhibits energy increase in higher wavenumbers. Proposed method works well although there exists spectral broadening since the spectra still satisfies the *M* = *O*(*K* log(*N*/*K*)) condition of the compressive sampling. As a numerical check; for a peak period of *T*_*p*_ = 12 *s* and significant wave height of *H*_*s*_ = 1 *m*, the *N* component Jonswap spectra decreases almost to zero at the 80*th* point of the wavenumber vector. Therefore for this case the two sided Jonswap spectra can be thought as 160-sparse function. Therefore for *N* = 1024 at least *M* = *O*(160 log(1024/160)) = *O*(129) sampling points are necessary. Although this requirement forces *M* to be bigger in the case of spectral broadening, the proposed method still performs significantly better in terms of computation time as discussed in the coming sections.

**Figure 15:**
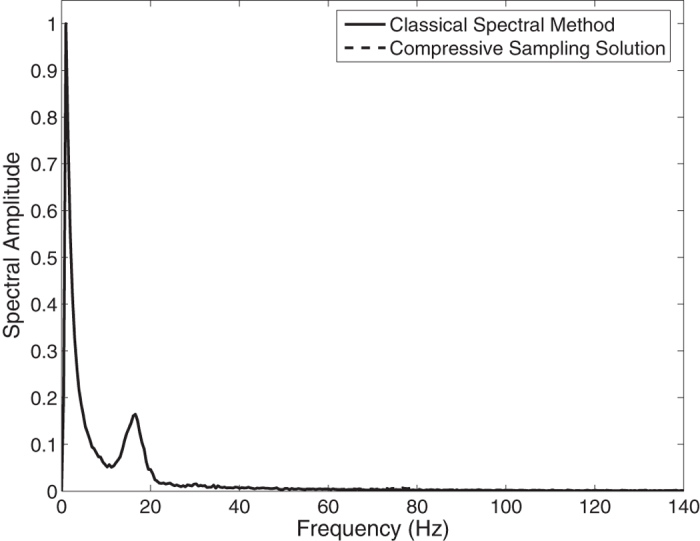
Comparison of classical spectral method and proposed compressive spectral method for a Jonswap spectrum with *N* = 2048, *M* = 256-two peaked spectra.

**Figure 16:**
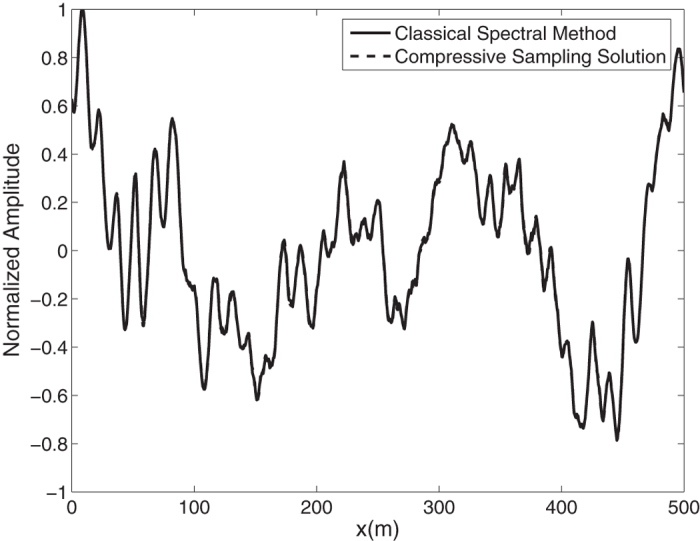
Comparison of classical spectral method and proposed compressive spectral method for a fully nonlinear periodic wave profile with *N* = 2048, *M* = 256-two peaked spectra.

As a further check on the spectral broadening, we consider a Jonswap type spectrum with energy concentrated around two peak periods initially. Such simulations can be beneficial for modeling shorter waves with more energy superimposed on longer wave trains as well as swell simulations. Again smoothing filter is applied in this simulation and a run time of 50*T*_*p*_ is used. As it can realized from the Figs 15 and 16, which show the spectra and water surface fluctuation respectively, the method also works well in the case of two peaked spectra. Although there exists spectral broadening, the spectra still satisfies the *M* = *O*(*K* log(*N*/*K*)) condition of the compressive sampling therefore proposed compressive spectral method works well. If longer runs are performed with various configurations higher values for *M* may be needed due on the restrictions on *M*. In that case the proposed compressive spectral method would be less efficient than itself with a smaller *M*, but still more efficient than the classical spectral method. However with the examples presented above it is shown the proposed compressive sampling method can accurately model the fully nonlinear ocean waves in the typical temporal and frequency range including the spectral broadening.

In reality, for example in very shallow water with many wave interactions and capillary components the spectrum would be too wide for the CS to make the exact recovery. So in that case since the sparsity property is not satisfies the proposed method would fail. However as the spectrum gets wider, the actual surface profile would get narrower and more sparse. Therefore in that case by means of a hybrid method it would be possible to switch to perform the CS in the physical domain by taking samples in the frequency domain. However this is a part of future study and not addressed in this paper.

All of the results presented above show promising evidence for the accuracy of the proposed method. Additionally the computational efforts required to run the various configurations are summarized in the Table 2. The average computation times of 50 realizations given in the table are in the units of seconds. The times are measured on a Dell Vostro 1700 laptop with dual core of 1.8 GHz and 1 GB RAM which is used to run the MATLAB code. As it can be seen on the table, for smaller number of time steps the compressive spectral method provides a small amount of improvement in the computational time. This is due to the computational effort required by the *l*_1_ minimization. However for the bigger number of time steps, the computational time is significantly improved while the differences in the wave profiles are of negligible importance. Therefore by reducing the computation time the compressive spectral method provides a great computational efficiency compared to the classical spectral method and can be used as a tool in not only for ocean wave simulations but also many other phenomena in applied mathematics and physics.

## Discussion

In this study compressive spectral method for the simulation of the linear and the fully nonlinear ocean gravity waves is introduced. The sparsity property of Jonswap frequency spectrum of the ocean waves is used for this purpose. It is shown that by using a smaller number of spectral components and the compressive sampling technique, it is possible to reconstruct the ocean surface with negligible difference in accuracy compared to the classical spectral method which uses a bigger number of spectral components. It is shown that the proposed compressive spectral method improves the computational effort significantly compared to the classical spectral method while still correctly models the nonlinear ocean waves including spectral broadening. This improvement becomes more significant especially for large time evolutions.

## Additional Information

**How to cite this article**: Bayındır, C. Compressive Spectral Method for the Simulation of the Nonlinear Gravity Waves. *Sci. Rep.* **6**, 22100; doi: 10.1038/srep22100 (2016).

## Figures and Tables

**Table 1:** Comparison of Temporal Cost of the Classical Spectral vs Proposed Method-Linear Wave Simulations.

*N*	*M*	# of Time Steps	Class. Meth.-Time (sec)	Prop. Meth.-Time (sec)	Normal Rms Diff.
1024	128	30000	61.90	8.91	0.0036
1024	256	70000	146.03	48.92	0.0052
1024	256	90000	189.31	55.89	0.0066
1024	256	120000	256.55	52.04	0.0070
2048	256	60000	341.09	232.16	0.0048

**Table 2:** Comparison of Temporal Cost of the Classical Spectral vs Proposed Compressive Spectral Method-Full Spectrum Simulations.

*N*	*M*	# of Time Steps	Class. Meth.-Time (sec)	Prop. Meth.-Time (sec)	Normal Rms Diff.
102400	128	20000	116.98	38.71	0.0140
102400	256	20000	161.34	135.38	0.0011
102400	256	60000	366.88	152.26	0.0037
102400	256	100000	618.92	132.13	0.0019
204800	256	50000	705.15	601.74	0.0077

## References

[b1] Bayindir C. *Implementation of a Computational Model for Random Directional Seas and Underwater Acoustics*. Master’s thesis, University of Delaware (2009).

[b2] Karjadi E. A., Badiey M. & Kirby J. T. Impact of surface gravity waves on high-frequency acoustic propagation in shallow water. J. Acoust. Soc. Am. 127, 1787–1787 (2010).

[b3] Karjadi E. A., Badiey M., Kirby J. T. & Bayindir C. The effects of surface gravity waves on high-frequency acoustic propagation in shallow water. IEEE J. Ocean. Eng. 37, 112–121 (2012).

[b4] Dommermuth D. G. & Yue D. K. P. A high-order spectral method for the study of nonlinear gravity waves. J. Fluid Mech. 184, 267–288 (1987).

[b5] Mei C. C., Stiassnie M. & Yue D. K. P. Theory and Applications of Ocean Surface Waves, Part 2: Nonlinear Aspects (World Scientific, Massachusetts, 2005).

[b6] Zakharov V. E. Stability of periodic waves of finite amplitude on the surface of a deep fluid. Sov. Phys. JETP 2, 190–194 (1968).

[b7] Demiray H. & Bayindir C. A note on the cylindrical solitary waves in an electron-acoustic plasma with vortex electron distribution. Phys. Plasmas 22, 092105 (2015).

[b8] Canuto C. Spectral Methods: Fundamentals in Single Domains (Springer-Verlag, Berlin, 2006).

[b9] Trefethen L. N. Spectral Methods in MATLAB (SIAM, Philadelphia, 2000).

[b10] Candès E. J., Romberg J. & Tao T. Robust uncertainty principles: Exact signal reconstruction from highly incomplete frequency information. IEEE Trans. Inf. Theory 52, 489–509 (2006).

[b11] Candès E. J. Compressive sampling. Proceed. Intern. Congress of Mathematicians 3, 1433–1452 (2006).

[b12] Bayindir C. Early detection of rogue waves by the wavelet transforms. Phys. Lett. A 380, 156–181 (2016).

[b13] Bayindir C. Shapes and statistics of the rogue waves generated by chaotic ocean current. *arXiv Preprint* **arXiv:1512.03584** (2015).

[b14] Bayindir C. Compressive split-step Fourier method. TWMS J. of Apl. & Eng. Math. 5, 298–306 (2015).

[b15] Mori N. & Yasuda T. Effects of high-order nonlinear interactions on unidirectional wave trains. Ocean Eng. 29, 1233–1245 (2002).

